# Transparency of Health-Apps for Trust and Decision Making

**DOI:** 10.2196/jmir.2981

**Published:** 2013-12-30

**Authors:** Urs-Vito Albrecht

**Affiliations:** ^1^PL Reichertz Institute for Medical InformaticsHannover Medical SchoolHannoverGermany

**Keywords:** smartphone, technology, education, medicine, app, health care, Android, iPhone, BlackBerry, Windows Phone, mobile phone, standards

Smart devices such as smartphones and tablet PCs have become an integral part of everyday life as well as for professional applications. This is also true for medicine [1]. To enhance patient safety for medical apps or health apps that are to be used successfully in today’s medical settings, a good information policy should always be part of the marketing strategy. Patients and doctors that are well informed about the benefits, limits, and risks of an app are in a better position to give more reasoning to their decisions whether they want to use it in a medical context or not.

To address the current shortcomings concerning the way information about apps is provided to potential users of apps, Lewis, in a recent letter to JMIR, proposed a set of standard criteria [2] analogous to those published by the Health on the Net foundation [3] to be used for assessing the utility of medical apps based on a systematic self-certification model. He suggested using a central platform for this purpose, for example, the United Kingdom National Health Service App Store, to allow registered developers of mobile medical apps to highlight the fact that they conform to these criteria. This would probably also give developers and distributors of such apps an advantage over their competitors.

While this certainly is a promising approach, I would like to add a few points. For one, in an international setting with users coming from various (and in many cases non-professional) backgrounds, it may be difficult to lead them to a separate platform that is not the standard app distribution platform that users are accustomed to. This is especially the case for casual users who probably tend to use information that is readily available on the app stores or to simply read what other users have to say about an app.

In my opinion, the users themselves should not be disregarded in the overall process since they play an important role by applying the information they have at hand to the product they are interested in and evaluating whether it meets their needs. In contrast to other medical products (eg, for clinical use), where many professional users are confronted with already chosen products that have been labeled as appropriate by experts, many professionals or laypersons have to decide on the appropriateness of the medical app themselves. Therefore, especially for apps, ensuring patient safety also has to include the identification of the right product, in this case an app, that matches the desired setting and indication. Every piece of information covering the necessary aspects helps decision makers and/or end users in professional settings as well as for private use to determine whether an app can be trusted and safe. Thus, to ensure high impact, it would probably make sense to provide users with the appropriate information at places where they expect it (ie, directly in an app’s description on the respective app store and/or on the manufacturer’s homepage and/or marketing material). This should be done following a standardized structure that includes criteria with a clear rationale (Table 1), for example, in the form of a clearly structured app synopsis (Table 2) [4]. A basis for this was proposed in [5], which also included the aspects mentioned in [2] with more detail.

There are already a number of existing initiatives and projects that use almost identical criteria to those suggested by Lewis, for example, the “Apps Peer-Review” by the Journal of Medical Internet Research (JMIR) launched in 2013 [6]. The JMIR mHealth disclosure form [7] also covers many of the concerns mentioned in the proposed app synopsis. Mostly, these projects reach this goal by installing certification and/or (third party) review processes and publishing the corresponding evaluation results using specific channels (eg, their own webpage or scientific journals). The app synopsis could be seen as a “first level” approach according to criteria already specified by previous projects dealing with quality assurance for Web-based information sources [8], though its focus is slightly different. At first, it could serve to provide all interested parties with sufficient information that, in addition to providing customers with basic information about an app, can then also be used as a starting point for building tests (eg, to identify the appropriate reviewers and testing methods, independent of the business model or revenue strategy that is employed by each respective initiative). The current market players come from different backgrounds and thus also have different interests in mind. In Germany, for example, there are some initiatives focusing mainly on a single disease while others target health apps in general. Also, their funding strategies differ significantly, ranging from privately funded initiatives or publicly financed institutions to companies that are being paid on a case-by-case basis.

If manufacturers were to publish the necessary information following this app synopsis, both they as well as the users would clearly benefit. Users would receive a complete and easily comprehensible set of information that would support them in their decision making process while manufacturers would be able to follow the simple structure of the synopsis to compile the necessary information without expending too much effort since they only have to compile information that should already be available to them. Although this is not equivalent to an officially sanctioned certification process, information published according to the synopsis could nevertheless serve as a reference if there are any disputes between both sides.

 

**Table 1:** Criteria for assessing health apps and medical apps.

Criteria	Content	Rationale
Imprint	Information about the manufacturer/distributor and associates	To get in touch, to identify conflicts of interest (influence) of the sponsor and all associated parties
	Metadata of the app	To get basic information about the actuality of the app
Rationale	Description of the app’s intended purpose(s), targeted user(s), the dedicated setting of the app, its categorization as a medical / non-medical app	To understand the idea behind the app, its categorization on a professional level and its ideal deployment setting and field of application
Functionality	Description on the functionalities and features of the app and the restrictions and limits	To understand the underlying functions to achieve the app's purpose(s) and its limits and risks to estimate whether the app is safe for usage
	Details about what measures have been taken to assure good usability of the app	To be informed about methods that were employed during the development cycle regarding the app’s usability for specific target groups
Validity and reliability	Description and reliability of information sources the app is based on	To assess whether the content and its authors are reliable and whether the functionality base on reliable and valid information sources
	Description of quality assurance methods	To estimate the level of quality in the production process of the app
Data requisitioning & management	Description of the amount and types of data that are being collected and processed	To be able to determine whether the app’s data collection & processing are adequate to fulfil the stated purpose
Data protection & privacy	Information about the manufacturer’s adherence to data protection and privacy laws and regulations and the involved jurisdictions	To find out whether the manufacturer provides a privacy statement and data protection policy that is well adapted to the app’s purpose
Data transmission & storage	Description of all measures taken to protect data entrusted to the app	To assess whether data transmission & storage is protected adequately

**Table 2:** Detailed description of items of the App-Synopsis for health apps and medical apps.

Item Category	Checklist Item	Sub Items
1. Imprint	1.1 Meta Data	1. Operating system
		2. Version number
		3. Web link (project pages and link to the app store)
		4. Category: Commercial project, non-commercial project, other
		5. Category: public access via an app store, only available to a restricted number of users/experts (in-house), other (please specify)
	1.2 Developer/Distributor	1. Information about the manufacturer/developer
		1.1 Name, address, webpage, contact person(s), email address, phone and fax number
		2. Information about the distributor
		2.1 Name, address, webpage, contact person(s), email address, phone and fax number
	1.3 Sponsoring/Advertising	1. Information about the funding used for developing the app
		1.1 Category: sponsoring, advertisements, other
2. Rationale	2.1 Category	1. Category: medical product or not, if yes: which class; has the app been certified voluntarily (by whom?), uncertified app
	2.2 User group	For each user group:
		1. Specific disease/condition (or as an alternative/addition: which health care professions are targeted, etc)
		2. Gender, age (range), other descriptive items
	2.3 Setting	1. Clinical, outpatient setting, at home, other
		2. Short description of a typical “use case”
	2.4 Purpose	1. Short description of the purpose of the app
		2. Category: information, reference work, educational resources, documentation, diagnostics, therapy, prevention, research, other
		3. Basic description of what the app is to be used for including specific information for the user group(s)
3. Functionality	3.1 Functions and Features	For each available function/feature:
		1. Function (designation)
		1.1 Example
		1.2 Source(s)
		1.3 Category: scientifically accepted, up-to-date content and reflects the current state of science and technology, evidence level if applicable
	3.2 Restrictions and Limits	1. Restrictions and limits of the app
		1.1 Specific description of the app’s restrictions and limits
		1.2 Description of potential or existing risks for the user group(s)
		1.3 Measures that have been implemented to avoid risks for the user group(s)
		2. Already known undesirable effects
		2.1 Detailed description of undesirable effects, if any
	3.3 Usability	1. Methods that were employed during the development cycle
		1.1 Results of usability testing
4. Validity and reliability	4.1 Content	1. Information about the expert(s) responsible for the app’s content
		1.1 Name of the author(s)
		1.2 Description of the qualification of the expert(s)
		1.3 Description of potential or actual conflict of interest
		2. Information about source(s)/reference(s) for all content and algorithms integrated into the app
		2.1 Specific information about the source(s)
		2.2 Evidence level of the source(s)
		3. Studies that have been performed concerning the app
		3.1 Type of the study, references/literature, other evidence
		4. Additional material about the app (test reports, etc)
		4.1 Type of additional material, reference links, ...
	4.2 Quality assurance	1. Information about quality assurance measures that were used during development
5. Data requisitioning & management	5.1 Data handling	1. Data processing
		1.1 Information about data collection mechanisms integrated into the app
		2. Data protection & privacy
		2.1 Voluntariness of participating in any data collection
		3. Data transmission & storage
		3.1 Purpose of the data collection
		3.2 Who profits from the collected data
		3.3 What kind of and how much data are being collected, at what times (including time intervals where applicable)? In which country is the data being stored? This is especially important considering the differences between data privacy laws and regulations in different countries.
		3.4 Which methods are being used for storing and evaluating the data?
		3.5 Specifics about user’s rights to obtain information about any data that are stored about him; in addition, there must be means to revoke an already given permission to store data. For this purpose, a contact address must be specified.
		3.6 It must also be possible to delete data that have already been stored and the user must be informed about the timespan that is needed until the data are really deleted.
		3.7 Encryption methods and level used for protecting the user’s data during transmission, storage and evaluation. It should also be specified whether it is possible to connect a specific user to the stored data or whether the data are being stored anonymously or pseudonymized.
		3.8 An indication about whether it is possible to prevent data collection and/or transmission and if yes, how this is possible.
